# Calling for policy actions to increase access to long-acting antipsychotics in low-income and middle-income countries

**DOI:** 10.1017/S2045796022000166

**Published:** 2022-05-11

**Authors:** Giovanni Ostuzzi, Chiara Gastaldon, Davide Papola, Corrado Barbui

**Affiliations:** Department of Neuroscience, Biomedicine and Movement Sciences, Section of Psychiatry, WHO Collaborating Centre for Research and Training in Mental Health and Service Evaluation, University of Verona, Verona, Italy

## Abstract

Schizophrenia-spectrum disorders are associated with substantial impairment and disability. Lack of treatment adherence is a major issue, especially in low- and middle-income countries (LMICs). Despite growing evidence supporting second-generation long-acting antipsychotics (LAIs) as an effective strategy to ensure continued maintenance treatment in schizophrenia, access to these technologies has been very limited in constrained-resource settings. Including second-generation LAIs in national and international essential medicines lists and evidence-based guidelines, promoting public health-oriented patent pooling and extending their availability to primary health care settings, are key actions that should urgently be implemented to increase access to long-acting technologies. Implementing these policy actions can pragmatically improve treatment adherence, ultimately tackling schizophrenia-related impairment and disability in LMICs, which can be regarded as a global health priority.

Mental disorders are a leading cause of years with lived disability. The Global Burden of Disease 2019 estimated that, from 1990 to 2019, the global number of disability-adjusted life years (DALYs) due to mental disorders increased from 80.8 million to 125.3 million, and the proportion contributed by mental disorders increased from 3.1 to 4.9% (GBD, 2022). Among mental disorders, the burden of schizophrenia is substantial, as measured in prevalence, disability-adjusted life-years and years lived with disability, particularly in low- and middle-income countries (LMICs) (Charlson *et al*., 2018). Financial and human resources invested in tackling this burden are insufficient, inequitably distributed and inefficiently used, resulting in a large treatment gap (WHO, 2021*a*).

Among other factors, lack of regular antipsychotic use in individuals with schizophrenia requiring maintenance treatment contributes to this gap, especially in LMICs (Chisholm *et al*., 2017). In these settings, regular antipsychotic use is often limited by several logistic and economic issues, including difficulties in offering and maintaining regular clinical follow-up, leading to a high risk of missing daily intake of medicines; prohibitive health-related costs to health systems and end-users, especially in populations with no financial protection or health insurance; and poor functioning supply chains, affecting regular availability of medicines, especially in rural areas (Barbui, 2015; Barbui *et al*., 2017; WHO, 2017). People living in remote locations with limited access to health care facilities might be further penalised during periods of local and global crisis, as observed during the severe acute respiratory syndrome coronavirus 2 (SARS-CoV2) pandemic (IASC, 2020; de Voursney *et al*., 2021). Furthermore, socio-cultural aspects, such as poor awareness of mental health problems, stigma and discrimination may negatively impact help-seeking behaviour (Barbui *et al*., 2016).

Current evidence show that long-acting antipsychotics (LAIs) can effectively address poor treatment adherence in individuals requiring long-term use (Kishimoto *et al*., 2021), and possibly reduce the risk of relapse in clinically stabilised individuals even after discontinuation as compared to their oral counterparts (Schoretsanitis *et al*., 2022). Some second-generation LAIs, including paliperidone, olanzapine and aripiprazole, showed the highest effect sizes and certainty of evidence for both relapse prevention and overall acceptability (Ostuzzi *et al*., 2021). For these reasons, increasing access to second-generation LAIs in LMICs may offer an opportunity to improve regular antipsychotic use in individuals with chronic schizophrenia (Ostuzzi and Barbui, 2021). Long-acting formulations have been already fully recognised in most LMIC to improve the prevention and treatment of malaria, tuberculosis, hepatitis C and HIV (Marmora *et al*., 2018). Recently, the Inter-Agency Standing Committee's COVID-19 guidance indicated long-acting formulations as a resource to maintain essential health services while reducing face-to-face visits during pandemic periods (IASC, 2020).

Expanding access to LAIs in LMICs would require to overcome several demand-side and supply-side barriers. Demand-side barriers include patients' and caregivers' perception of LAIs as a possibly coercive tool, particularly if previous traumatic experiences of coercion occurred (Taylor *et al*., 2018). A similar perception may be culturally rooted also in prescribers, who may consider these formulations only as a last resort for chronically ill individuals (Lindenmayer *et al*., 2020). In terms of supply-side factors, access in LMICs is limited by the lack of inclusion of any LAIs from some national essential medicines lists (EMLs), and by the exclusion of second-generation formulations from most national EMLs. We calculated that, of 107 national EMLs stored in the WHO repository of National Medicines List/Formulary/Standard Treatment Guidelines, 16 (15%) do not include any long-acting formulations, and 94 (88%) do not include a second-generation LAI (22/24, 92%, in low-income countries; 72/83, 87%, in middle-income countries). These figures are worrisome, as inclusion of a particular medicine in national EMLs has been shown to result in higher availability, particularly in the public sector and in LMICs (Bazargani *et al*., 2014). Additional supply-side impediments may include generic availability issues such as weak procurement and distribution chains, and affordability issues such as high costs to health systems and to end users of formulations that may still be protected by patents or exclusivities.

Against this background, here we suggest some policy actions to enhance the capacity of LMICs to increase access to LAIs, including second-generation formulations. As recommended by a recent thematic paper commissioned by the Gulbenkian Global Mental Health Platform and the WHO, actions may be organised as a function of the four components of access: selection, availability, affordability and appropriate use (Barbui *et al*., 2016; WHO, 2017).

A first action is that decision-makers in LMICs should re-examine national EMLs in view of the growing evidence in support to the efficacy of second-generation LAIs. As the addition of new medicines to national EMLs is guided by the WHO EML, a model list of medicines considered essential for basic health-care needs (Wirtz *et al*., 2017), it is important that this model list is kept updated with current best evidence. The 21^st^ WHO EML used to include only fluphenazine decanoate, a first-generation LAI whose provision is erratic because of production and supply problems. We therefore drafted an application for the 23^rd^ WHO Expert Committee on Selection and Use of Essential Medicines, which convened in Geneva on 21 June to 2 July 2021. The application was based on the results of a network meta-analysis on relapse prevention and acceptability of LAIs in the maintenance treatment of adults with schizophrenia-spectrum disorders (Ostuzzi *et al*., 2021). The review found most LAIs to be similarly effective and acceptable, although paliperidone palmitate (1 and 3-monthly formulation), olanzapine and aripiprazole showed the highest effect sizes and certainty of evidence for both relapse prevention and overall acceptability. Based on this application, the WHO decided to include in the WHO EML paliperidone palmitate 1-monthly formulation with a square box indicating risperidone long-acting injection as a therapeutic alternative (WHO, 2021*c*). Paliperidone palmitate was selected for several reasons. As paliperidone is a metabolite of risperidone, and oral risperidone is already included in the WHO EML, people on maintenance treatment with oral risperidone may easily switch to paliperidone palmitate (Schoretsanitis *et al*., 2018). Further, as opposed to risperidone microspheres long-acting, paliperidone palmitate does not require refrigeration, and allows a longer interval between injections, which is of practical relevance for constrained-resource settings. Likely, the inclusion of long-acting formulations of paliperidone and risperidone in the WHO EML will encourage countries to add a representative of second-generation LAIs to national EMLs.

A second action refers to measures aiming to increase availability. In addition to generic measures that are not specific to any particular group of medicines, such as the development of a functioning and reliable supply system, there are important measures that may profoundly affect the degree of availability of LAIs. For example, whether only doctors or also other professionals can prescribe these antipsychotic formulations, including initial and subsequent prescriptions, is a key policy aspect that decision-makers should address. Related to this, the level of the health care system and the conditions under which these medicines may be prescribed are similarly important, as medicines only offered in selected secondary and tertiary health facilities are usually poorly available in several LMICs. In countries with less extensive mental health coverage LAIs should probably be made available in primary health care settings, where mental health is usually integrated. In countries with a more developed mental health care system, LAIs should be made available in community mental health care services, which should implement outreach initiatives to increase access to treatment for people with severe mental disorders.

A third action refers to affordability measures. Pursuant to the strategy outlined by the WHO and the Lancet Commission on Essential Medicines Policies, we argue that second-generation LAIs should be included in the list of patented essential medicines to undergo public health-oriented patent pooling. This would support generic manufacturing, which is a valuable public health approach to improve accessibility of otherwise expensive treatments in LMICs (Burrone *et al*., 2019). Further, it would be important to include mental health treatment and medicines, including second-generation antipsychotics, in benefit packages under reimbursement systems, in countries where such systems exist.

Given their efficacy, tolerability and cost-effectiveness (Achilla and McCrone, 2013; Raghavan *et al*., 2020; Ostuzzi *et al*., 2021), LAIs might be suitable for inclusion in scaled-up universal health coverage programs for schizophrenia, whose development is a global mental health priority (Patel, 2016).

Once selected and included in national EMLs, and available and affordable to end users, LAIs should be appropriately prescribed. A fourth action is therefore required by national and international organisations to update existing treatment guidelines, emphasising the need for an earlier and broader use of LAIs, especially second-generation formulations. These formulations should be offered as an alternative to oral antipsychotics when poor treatment adherence is a clinical priority and when regular scrutiny of mental health and adverse effects is needed, rather than as a ‘last resort’ for the most stigmatised and chronically ill individuals. Aiming to overcome the cultural stigma surrounding their use, guidelines should emphasise the importance of explicitly discussing benefits and harms with individuals suffering from schizophrenia and their family members, actively involving them in the choice of treatment (Hui *et al*., 2019).

We argue that policy actions to prioritise access to LAIs in LMICs should be carried out, as these medications might represent a key tool to expand access to mental healthcare globally. Mental Health Atlas 2020 reported that service coverage for psychosis is extremely low, with a global median of 29% of individuals with psychosis receiving mental health services, with wide differences by country income level (WHO, 2021*b*). As increasing service coverage at least by half is one of the core targets of the WHO Comprehensive Mental Health Action Plan 2013–2030 (WHO, 2021*a*), we suggest that optimising access to LAIs may give the chance for a transformative improvement of the whole mental healthcare system, offering a unique opportunity to reach this ambitious core target.

## Data

Not applicable, as no original data have been analysed to write this editorial.
